# Evolution of the Crop Rhizosphere: Impact of Domestication on Root Exudates in Tetraploid Wheat (*Triticum turgidum* L.)

**DOI:** 10.3389/fpls.2017.02124

**Published:** 2017-12-13

**Authors:** Anna Iannucci, Mariagiovanna Fragasso, Romina Beleggia, Franca Nigro, Roberto Papa

**Affiliations:** ^1^Centro di Ricerca per la Cerealicoltura e le Colture Industriali, Consiglio per la Ricerca in Agricoltura e l'analisi dell'Economia Agraria (CREA-CI), Foggia, Italy; ^2^Dipartimento di Scienze Agrarie, Alimentari e Ambientali, Università Politecnica delle Marche, Ancona, Italy

**Keywords:** domestication, root exudates, wheat, tetraploid wheat, *Triticum turgidum*, metabolites, rhizosphere

## Abstract

Domestication has induced major genetic changes in crop plants to satisfy human needs and as a consequence of adaptation to agroecosystems. This adaptation might have affected root exudate composition, which can influence the interactions in the rhizosphere. Here, using two different soil types (sand, soil), we provide an original example of the impact of domestication and crop evolution on root exudate composition through metabolite profiling of root exudates for a panel of 10 wheat genotypes that correspond to the key steps in domestication of tetraploid wheat (wild emmer, emmer, durum wheat). Our data show that soil type can dramatically affect the composition of root exudates in the rhizosphere. Moreover, the composition of the rhizosphere metabolites is associated with differences among the genotypes of the wheat domestication groups, as seen by the high heritability of some of the metabolites. Overall, we show that domestication and breeding have had major effects on root exudates in the rhizosphere, which suggests the adaptive nature of these changes.

## Introduction

Domestication has shaped the genome of crop plants to satisfy human needs for adaptation to agroecosystems. Recent studies have shown that this process had profound phenotypic consequences far beyond the few traits of the domestication syndrome, which also involved genome-wide transcriptomic and metabolomic changes (Bellucci et al., 2014; Beleggia et al., 2016). Considering the crucial role of root-system and rhizosphere interactions for plant adaptation (in relation to the major changes in soil management associated with cultivation), one of the major questions is whether and how domestication and breeding have had any impact on these phenomena (Bulgarelli et al., 2015; Gioia et al., 2015; Kuijken et al., 2015). Indeed, a series of complex chemical, physical and biological interactions takes place between roots and their surrounding environment. Plants contribute to these interactions by secretion of an enormous range of metabolites from their roots into the surrounding soil (Badri and Vivanco, 2009). These root exudates are generally classified into two classes of metabolites: high molecular weight exudates, which contain polysaccharides and proteins; and low molecular weight metabolites, which include amino acids, organic acids, sugars, phenolics, and various secondary metabolites (Badri and Vivanco, 2009).

Rhizosphere biology has recognized the biological importance of root exudates in the mediation of interactions with other plants and with microbes, in terms of competing roots and pathogenic and non-pathogenic microbes and invertebrates (Pangesti et al., 2013). Hence, root exudates provide a source of allelochemicals that can mediate plant-to-plant interactions (Fragasso et al., 2013), and as such, they represent chemical information that can influence vital physiological processes (e.g., respiration, protein biosynthesis, photosynthesis, cell division and elongation) (Field et al., 2007). At the same time, these allelochemicals provide the molecular basis for plant–microbe interactions in the rhizosphere (Bais et al., 2006) that are responsible for plant health and growth (e.g., defense against diseases, facilitation of nutrient acquisition) (Pérez-Jaramillo et al., 2016). The biological significance of these processes helps us to understand why plants sustain significant carbon costs to maintain the processes of root exudation (Uren, 2007). Furthermore, in *Arabidopsis thaliana*, possible connections have been highlighted between changes in root architecture and plant-influenced chemical changes in the rhizosphere microbiome diversity (Micallef et al., 2009).

Dissimilarities in root architecture between modern varieties and their wild relatives have been reported for several crops (Pérez-Jaramillo et al., 2016), and recently, Gioia et al. (2015) described the impact of domestication of tetraploid wheat on its shoot and root phenotypic architecture. Increasing evidence indicates the potential influence of soil type on growth investment and rhizosphere-associated metabolites (Kuijken et al., 2015). At the same time, several indications suggest that different genotypes of the same species can promote different rhizosphere compositions (Bulgarelli et al., 2015).

Root exudates have a major role in the mobilization of soluble nutrients in the rhizosphere (Carvalhais et al., 2011), and the release into the rhizosphere of diverse organic materials that can influence the soil structure (Traoré et al., 2000). In addition, root exudates contain allelochemicals of biological significance to the rhizosphere (Bertin et al., 2003; Iannucci et al., 2013), and increasing evidence suggests that root exudates initiate and modulate the dialog between roots and soil microbes (Badri and Vivanco, 2009). A major question here is thus how much different plant genotypes and populations contribute to genetic differences in root exudate composition, as recently shown in *Arabidopsis* (Mönchgesang et al., 2016), and whether these differences have any adaptive value, and hence potential to be exploited for plant breeding (Fernández-Aparicio et al., 2014; Kuijken et al., 2015).

One approach to address this aspect is based on the analysis of different genotypes and soil types, with direct measurements of soil fertility, microbiome composition, and plant growth (Micallef et al., 2009). However, this direct approach can be very time consuming, and it can be complicated by a lack of knowledge of the strategy needed to select the plant genotypes to conduct such experiments. Here, we have adopted an evolutionary approach that is based on characterisation of the root exudates of a small panel of genotypes that were sampled on the basis of an evolutionary transect that spans the entire diversity of tetraploid wheat, and that has been used previously for analysis of root and shoot growth, and for evolutionary metabolomics in wheat (Gioia et al., 2015; Beleggia et al., 2016). The aim was thus to answer two main questions:

1. Are there differences in root exudate composition that suggest genetic diversity of root exudates that is potentially relevant in the conditioning of the rhizosphere composition?
2. If there is indeed this diversity, are there any differences that suggest differential adaptation between subspecies that are known to have undergone different selection pressures associated with the diverse intensification between wild early wheat domesticates and modern durum wheat?

In particular, we analyzed the metabolite variations in the rhizosphere soil across 10 wheat genotypes. These were chosen to represent three taxa that correspond to the key steps in the domestication of tetraploid wheat: primary domestication of emmer (*Triticum turgidum* ssp. *dicoccum*) from wild emmer (*Triticum turgidum* ssp. *dicoccoides*), and secondary domestication and development of durum wheat (*Triticum turgidum* ssp. *durum*) from domesticated emmer.

## Materials and methods

### Plant growth and soil sampling

**Figure 1:**
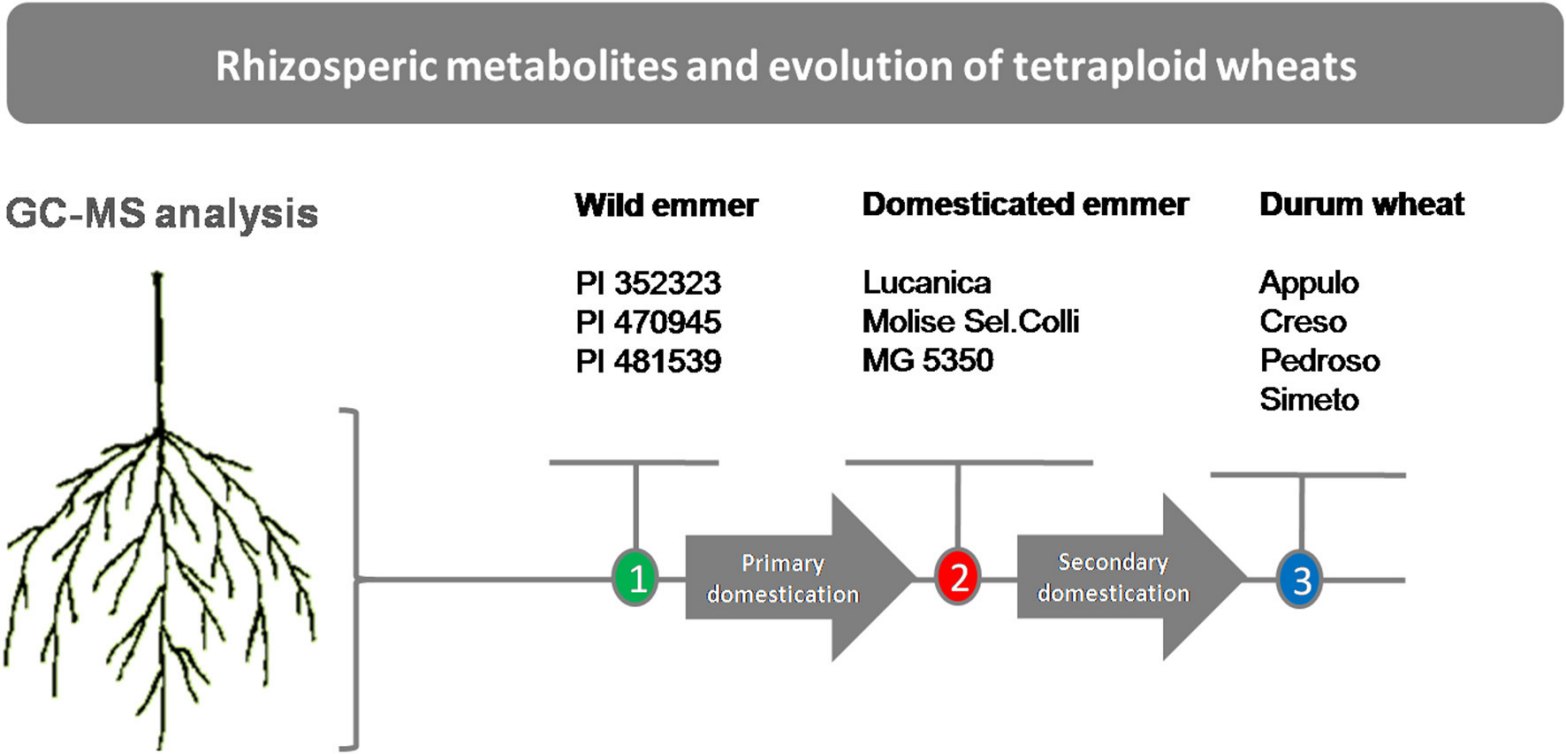
Schematic representation of the experimental plan. Wild emmer (*T. turgidum* ssp. *dicoccoides*), domesticated emmer (*T. turgidum* ssp. *dicoccum*), durum wheat (*T. turgidum* ssp. *durum*).

Plant materials of the 10 genotypes that represent three stages in tetraploid wheat evolution (i.e., the domestication groups) were used in this study: three wild emmer lines (*T. turgidum* ssp. *dicoccoides*), referred to here as “wild emmer” (PI 352323, PI 470945, PI 481539); three primitive emmer domestic lines (*T. turgidum* ssp. *dicoccum*) referred to here as “emmer” (“Lucanica,” “Molise Colli,” “MG 5350”); and four modern durum wheat varieties (*T. turgidum* ssp. *durum*) referred to here as “durum wheat” (“Appulo,” “Creso,” “Pedroso,” and “Simeto”) (Figure 1). Although *T. turgidum* is an autogamous species, to avoid any residual within-accession heterogeneity, each accession was purified through two cycles of single-seed descent under controlled selfing conditions. Thus, each accession consisted of an inbred line.

These accessions were shared with the studies of Beleggia et al. (2016) and Gioia et al. (2015), which both investigated durum wheat domestication. They were obtained from a large collection that has been developed by the *Consiglio per la Ricerca in Agricoltura e l'Analisi dell'Economia Agraria, Centro di Ricerca per la Cerealicoltura* (CREA-CER, Foggia, Italy), and they have been characterized by a large number of molecular markers (diversity arrays technology, simple sequence repeats) and phenotypic traits (Laidò et al., 2013, 2014).

Two experimental substrates were used for the plant growth: (i) non-sterile soil/ sand mix, as soil:sand (50:50; v/v, which is here referred to as Soil50; and (ii) sterile sand (sterilized in an oven at 180°C for 1 h), which is here referred to as Sand100. The choice of soil50 was based on preliminary experiments to determine the best results of root development when carried out with different soil/sand mixtures. Before the pot experiments, in July 2013, soil with a history of exposure to annual cereal species was collected from the farms of the CREA-CER (Foggia, Italy) (41°28′ N, 15°34′ E, 76 m a.s.l.). Samples were collected from the upper 30 cm of the soil profile, air dried for 1 week, thoroughly mixed, passed through a 2 mm sieve to remove gravel fragments, cleaned of plant debris, and stored in a cold room (4°C) until further use. The soil was an non-sterilized loam soil (USDA classification system), with the following characteristics: 21% clay, 43% silt, 36% sand, pH 8 (in H_2_O), 15 mg kg^−1^ available P (Olsen method), 800 mg kg^−1^ exchangeable K (NH_4_Ac), and 21 g kg^−1^ organic matter (Walkey–Black method). Silica sand with a grain size from 0.4 mm to 0.1 mm was also used.

Before sowing, the seeds were surface sterilized by soaking in 2% sodium hypochlorite for 5 min, and then rinsed several times with distilled water. The seeds were transferred to Petri dishes with one sheet of Whatman No.1 filter paper that was moistened with 5 mL distilled water, and they were then kept in a dark incubator at constant 20°C for 48 h. Fifteen pre-germinated seeds of each genotype were seeded into each plastic pot (diameter, 7 cm; height, 26 cm) that contained either 1.3 kg Soil50 or 1.6 kg Sand100. These pots were lined with Whatman 3 MM filter paper to avoid soil loss.

Immediately after sowing, 200 mL and 150 mL deionised water were added to the Soil50 and Sand100 pots, respectively. To maintain the moisture, the seedlings were regularly watered to 70% of field capacity at 3-day intervals, with Hogland nutritive solution, which contained the following mineral nutrients (per L): Ca(NO_3_)_2_·4H_2_O (940 mg), MgSO_4_·7H_2_O (520 mg), KNO_3_ (660 mg), NH_4_H_2_PO_4_ (120 mg), sequestrene 330 Fe (70 mg), H_3_BO_3_ (2.8 mg), MnSO_4_·4H_2_O (3.4 mg), CuSO_4_·4H_2_O (0.01 mg), ZnSO_4_·4H_2_O (0.02 mg), and Na_2_MoO_4_·2H_2_O (0.01 mg). The pots were placed in a growth chamber with a 16/8 h light/ dark cycle at 20/16°C, with light of 1,000 μE m^−2^ s^−1^ photoactive radiation at the leaf surface. The experiments were performed using a completely randomized design, with six replicates. Six pots with no seeds were included as controls.

To determine whether there are differences in the root exudates between tetraploid wheats already at the early stages of development, the plants were grown until the third leaf developmental stage. They were then collected by pulling them from the soil in the pots, with all of the plant material manually removed from the pots. The roots were carefully separated and shaken gently to collect the root-zone, or rhizosphere, soil, excluding the roots. Immediately after each collection, the rhizosphere soil samples were placed at −80°C for at least 8 h, and then freeze dried. Finally, these lyophilised samples were kept at −20°C until analysis. To determine the dry biomass production, the shoot and roots from each pot were oven dried at 70°C for 72 h. The parameters measured are given as shoot dry weight (SDW; mg plant^−1^) and root dry weight (RDW; mg plant^−1^).

### Gas chromatography–mass spectrometry analysis of the rhizosphere soil

The freeze dried samples were milled (Pulverisette 7 Planetary Micro Mill; Classic Line, Fritsch) with an agate jar and balls, and stored at −20°C until analysis. Two grams of each rhizosphere soil sample were extracted with a mixture of methanol: water: chloroform (1:1:3; v/v/v), and stored for 30 min at 4°C. The samples were then centrifuged (4,000 × g, 10 min, 4°C) and aliquots of 1 mL of the polar phase and 1.5 mL of the non-polar phase from the extractions were dried under vacuum (Speedvac) for further analysis. The polar residues were dissolved and derivatised in methoxyamine hydrochloride in pyridine (70 μL, 20 μg mL^−1^) for 90 min at 37°C, and then incubated with N-methyl-N-trimethylsilyl-trifluoroacetamide (MSTFA; 120 μL) at 37°C for 30 min. The non-polar fraction was dissolved and derivatised with MSTFA (70 μL) at 37°C for 30 min. Polar and non-polar metabolites were analyzed using gas chromatography (Agilent 6890N; Agilent Technologies, USA) coupled with quadrupole mass spectrometry (Agilent 5975; Agilent Technologies, USA), as described by Beleggia et al. (2013). Briefly, the samples (1 μL) were injected in splitless mode, with the gas chromatography separation on an HP-5ms capillary column (60 m, 0.25 mm i.d.; film thickness, 0.25 mm). Helium was used as the carrier gas, at a constant flow rate of 1 mL min^−1^. For analysis of polar metabolites, the injection temperature, transfer line and ion source were set at 280°C, and the quadrupole was adjusted to 180°C. The oven was kept at 70°C for 1 min, then the temperature was increased at 5°C min^−1^ to 310°C, and held for 15 min. Subsequently, the temperature was increased to 340°C, and held for 1 min. The mass spectrometer was operated in electron-impact mode at 70 eV, and the scan range was from 30 to 700 amu.

The non-polar metabolites were analyzed as above, with minor modifications: the injection temperature and the transfer line were set at 250°C; the oven was kept at 70°C for 5 min, and then the temperature was increased at 5°C min^−1^ to 310°C, and held for 1 min.

The metabolites were identified through comparisons of the mass spectrometry data with those of the National Institute of Standards and Technology (NIST Chemistry WebBook)^1^ database and a custom library obtained with reference compounds. The gas chromatography–mass spectrometry quantification was performed using the Chemstation programme. The standards and all of the chemicals used were HPLC grade, and were from Sigma-Aldrich Chemical Co. (Deisenhofen, Germany); MSTFA was from Fluka.

On the basis of the data obtained for the controls, the metabolite contents were corrected by subtraction of the concentrations defined for the controls pots. Using the gas chromatography–mass spectrometry approach, comprehensive profiles of the components were obtained directly from crude exudates without sample fractionation. Overall, the sugar, organic acid, fatty acid, polyalcohol, and polycosanol contents were monitored in the rhizosphere soil.

### Statistical analysis

Six biological replicates from each treatment were used, and in addition to the morphological characteristics (i.e., SDW, RDW), the content of the individual metabolites and their classes were considered as quantitative traits for the statistical analysis. The data were examined for normality of distribution and homogeneity of variance, and analysis of variance (ANOVA) was used to determine the differences among the *Triticum* species. Mean discrimination was performed by applying Tukey's tests, with statistically significant differences determined at the probability level of *P* ≤ 0.05. To exclude the possibility that the variations in the levels of the classes of metabolites among the three taxa were due to correlation to the morphological traits, these were included as covariates in the analysis of variance (ANCOVA).

To determine the heritability and Q_st_ of each of the metabolites for both of the substrates (i.e., Soil50, Sand100), nested analysis of variance (NANOVA) was performed using the restricted maximum likelihood procedure and considering the two datasets separately, as used by Beleggia et al. (2016). For each quantitative trait (metabolite), this analysis allowed partitioning of the total variance (σ^2^ TP) into genetic variance component due differences between taxa (σ^2^ B), between genotypes within taxa (σ^2^ W), and to error/ environment (arising among individuals of the same genotype; i.e., among replicates) (σ^2^ e). Error/environmental variance (σ^2^ e) was taken away from the total phenotypic variance (σ^2^ TP = σ ^2^ e + σ ^2^ W + σ ^2^ B) to determine the total genetic variance (σ^2^ TG = σ ^2^B + σ ^2^W). We then calculated the heritability of each quantitative (metabolite level) trait as h^2^ = σ ^2^ TG / σ ^2^TP. To obtain a general comprehensive characterisation of the samples, the metabolites of the root exudates detected in each substrate separately (Soil50, Sand100) and combined (Soil50 + Sand100) were subjected to principal component analysis (PCA) based on correlations. Here, in contrast to Beleggia et al. (2016), Q_st_ was not used (in comparison to F_st_ from molecular data) to detect the signature of selection, as the sample size was too limited for such analysis. However, Q_st_ was used to estimate the proportion of variance due to differences between domestication groups might have originated not only by selection but also by drift due to population subdivisions that occurred during domestication. The first and second principal component axis scores were plotted to aid visualization of the species differences. All of the statistical analyses were carried out using the JMP software (version 8.0; SAS Institute Inc., Cary, NC, USA).

## Results

### Morphological characterization

**Figure 2:**
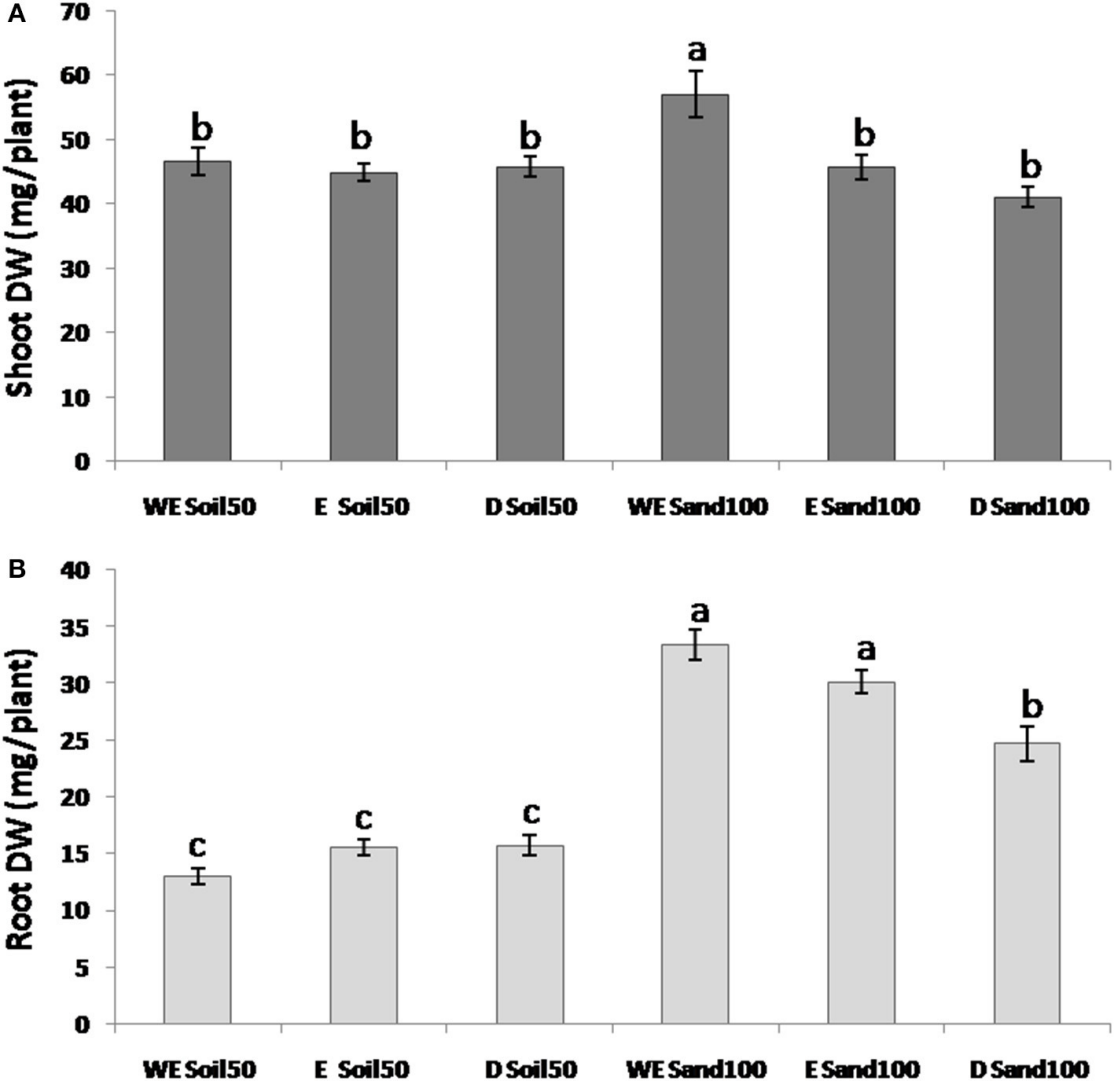
Morphological traits across the three different wheat species. **(A)** Shoot dry weight. **(B)** Root dry weight. WE, wild emmer; E, emmer; D, durum wheat.

For SDW, the ANOVA defined significant differences among the domestication groups and due to interactions between species and substrate (Figure 2A). Indeed, while with the Soil50 substrate the differences between species for SDW were not significant, with Sand100, wild emmer showed significantly higher SDW compared to both the emmer and durum wheat genotypes.

For RDW, all of the effects (i.e., substrate, subspecies, and their interaction) were highly significant. With the Sand100 substrate, RDW was significantly higher compared to plants grown with Soil50: 2.6-fold higher for wild emmer, 1.9-fold for emmer, and 1.6-fold for durum wheat. As for SDW, the RDW differences among the domestication groups were not significant with Soil50, while there were significant differences among the domestication groups with Sand100. In particular, wild emmer and emmer showed significantly higher RDW (33.4, 30.1 mg plant^−1^, respectively) compared to durum wheat (24.7 mg plant^−1^) (Figure 2B). Overall, wild emmer appears to be more plastic in terms of its increases in shoot growth (i.e., SDW) with the Sand100 substrate. For RDW, both wild emmer and emmer showed larger changes compared to durum wheat.

### Gas chromatography–mass spectrometry metabolite profiling

Overall, 17 different metabolites were detected in the root exudates (all 17 in Soil50 and 10 out of 17 in Sand100), all shared with previous work on tetraploid wheat kernels where 51 different metabolites were found (Beleggia et al., 2016). Analysis of the substrate of the controls (i.e., pots with substrate but no plants) with both Soil50 and Sand100 indicated that only seven and three compounds were detectable, respectively. Moreover, in both cases, these compounds were found at low concentrations (Table S1), as they were at least one order of magnitude lower than the levels observed in the presence of the plants. Thus, the observed variations in the metabolites across the genotypes can be considered to arise either as a direct consequence of the effects of the plant genotypes, or as the combined effects of the plants on the microorganism activities; i.e., as a consequence of the interactions in the rhizosphere.

To take in consideration the possibility that the root architecture had effects on the content of the metabolite exudates among the three taxa, the correlation between the morphological parameters (i.e., RDW, SDW) and the levels of metabolites were considered, including the morphological traits as covariates in the analysis of variance (ANCOVA). With the Soil50 trial, all of the metabolites showed greater abundance compared to the Sand100 substrate (mean, 32.2-fold greater) (Table 1). This was consistent among the different metabolite classes, although they showed different ratios, as 1.8-, 8.5-, and 33.4-fold for the polyalcohols, organic acids and sugars, respectively. The polycosanols were only detected with Soil50. Among the categories of metabolites detected with the Soil50 trial, the most abundant were the sugars, followed by the polyalcohols, organic acids, fatty acids, and polycosanols (Table 1). Indeed, all of the metabolite concentrations were significantly greater with Soil50 compared to Sand100. In contrast, with the exception of the polyalcohols, no significant effects were detected among the three subspecies or due to the interactions between the substrates and the subspecies. For the polyalcohols, both the treatments and their interaction were significant (T ≤ 0.0001; S ≤ 0.0287; TXS ≤ 0.0001). In particular, for this class of metabolite, with Soil50, the wild emmer showed significantly higher content compared to durum wheat, while with Sand100, durum wheat showed significantly higher content compared to both wild emmer and emmer (Table 1). Only the polycosanols showed a significant effect of the covariates, although without any changes compared to the ANOVA model without covariates (*P* ≤ 0.05).

**Table 1:** Concentrations of the metabolite classes in the rhizosphere according to Soil50 and Sand100 conditions for the wild emmer, domesticated emmer and durum wheat species, with harvesting at the third leaf developmental stage.

**Growth**	**Wheat**	**Metabolite class (ng g**^**−1**^**)**							
**Substrate**	**Species**	**Polar**			**Apolar**		**Polar**	**Apolar**	**Total**
		**Sugars**	**Poly alcohols**	**Organic acids**	**Fatty acids**	**Polycosanols**	**Total**	**Total**	
Soil50	Wild emmer	744741 a	1774 a	1070 a	1126.4 a	376.5 b	747585 a	1503.5 b	749088.5 a
	Emmer	721358 a	1447 ab	1189 a	1416.5 a	433.9 b	723994 a	1850.9 ab	725844.9 a
	Durum wheat	817845 a	1116 bc	1044 a	1378.6 a	569.4 a	820005 a	1948.0 a	821953.0 a
Sand100	Wild emmer	24785 b	751 cd	128 b	0.0 b	0.0 c	25664 b	0.0 c	25664.0 b
	Emmer	16773 b	435 d	91 b	0.0 b	0.0 c	17299 b	0.0 c	17299.0 b
	Durum wheat	26915 b	1162 bc	168 b	0.0 b	0.0 c	28246 b	0.0 c	28246.0 b
Soil50	Mean	761314	1446	1101	1307	460.0	763861	1822	765683
Sand100	Mean	22825	782	129	-	-	23736	.	23736
Soil50/ Sand100	Ratio	33.4	1.8	8.5	NA	NA	32.2	NA	32.2

*Data within the columns with different letters are significantly different (Tukey tests; P < 0.05)*.

Comparing the Soil50 and Sand100 treatments for differences in the individual metabolites in the rhizosphere soil, with the exceptions of ribose and mannitol, respectively, which showed no and minor differences, for all of the other metabolites here, significantly higher contents were generally detected with Soil50, which gave ratios with the Sand100 contents from 0.73 for ribose, to 47.7 for maltose and turanose (Table 2). In particular, with Soil50, the three domestication groups showed different patterns within the profiles of the rhizosphere metabolites (Table 2). Durum wheat was characterized by significantly higher contents of isomaltose, sucrose, hexadecanoic acid, octadecanoic acid and 1-octacosanol. Emmer and wild emmer both showed the highest contents of fructose, galactose and *myo*-inositol. Furthermore, emmer showed the highest concentrations of glycerol, mannose and glucose, while similar trends were seen in wild emmer for mannitol and sorbitol. There were no significant differences for the raffinose, ribose, maltose and turanose, oxalic acid, and 9-octadecenoic acid concentrations among the domestication groups. With Sand100, there were fewer metabolites in comparison to Soil50, and for those identified, the differences among the three domestication groups were attenuated (Table 2). Wild emmer was characterized by a high concentration of fructose, while the most abundant rhizosphere metabolites from durum wheat were mannitol and sorbitol. Intriguingly, for these three metabolites (i.e., fructose, mannitol, sorbitol), the Soil50 vs. Sand100 substrates changed rank order of their concentrations among the domestication groups (Table 2): for fructose: wild emmer = emmer > durum wheat with Soil50, and wild emmer > durum wheat > emmer with Sand100; for mannitol and sorbitol: wild emmer > emmer > durum wheat with Soil50, and durum wheat > wild emmer > emmer with Sand100.

**Table 2:** Concentrations of individual metabolites in the rhizosphere according to the Soil50 and Sand100 conditions for the wild emmer, domesticated emmer and durum wheat species, with harvesting at the third leaf developmental stage.

**Growth**	**Wheat**	**Metabolite (ng g**^**−1**^**)**																
**substrate**	**species**	**Fructose**	**Galactose**	**Isomaltose**	**Maltose + turanose**	**Mannose + glucose**	**Raffinose**	**Ribose**	**Sucrose**	**Glycerol**	**Mannitol**	***myo*-Inositol**	**Sorbitol**	**Oxalic acid**	**Hexadec acid**	**Octadec acid**	**9-Octadec acid**	**1-Octacosan**
Soil50	Wild	1075.6 a	324 a	87.9 ab	684491.5 a	2429.4 b	1674 a	52.9 a	54605.6 b	368.2 b	751.9 a	67.1 a	586.6 a	1070.1 a	60.7 b	751.4 b	314.3 a	376.5 b
	Emmer	1163.9 a	422.9 a	72.1 b	642497.6 a	3186.9 a	2262.2 a	56.7 a	71695.6 ab	443.1 a	533.1 ab	71.8 a	399.4 ab	1189.0 a	75.9 ab	820.1 ab	520.5 a	433.9 b
	Durum	624.1 b	164.7 b	121.6 a	734879.9 a	971.0 c	1957.4 a	37.9 a	79088.1 a	305.3 b	422.7 bc	29.3 b	358.5 b	1043.7 a	103.8 a	940.4 a	334.4 a	569.4 a
Sand100	Wild	331.5 c	143.5 b	0.00	11862.8 b	926.7 c	0.0 b	88.3 a	11375.4 c	15.0 c	339.7 bc	0.0 c	336.0 bc	128.1 b	0.0 c	0.0 c	0.0 b	0.0 c
	Emmer	79.2 d	65.4 b	0.00	9194.2 b	409.6 c	0.0 b	30.1 a	6994.9 c	3.5 c	271.5 c	0.0 c	159.7c	91.4 b	0.0 c	0.0 c	0.0 b	0.0 c
	Durum	247.8 cd	61.8 b	0.00	22166.7 b	762 c	0.0 b	84.0 a	3560.4 c	33.1 c	613.3 ab	0.0 c	515.3 ab	168.7 b	0.0 c	0.0 c	0.0 b	0.0 c
Soil50	Mean	954.5	303.9	93.9	687289.7	2195.8	1964.5	49.2	50443.4	372.2	569.2	56.1	448.2	1100.9	80.1	837.3	389.7	459.9
Sand100	Mean	219.5	90.2	0	14407.9	699.4	0	67.5	7310.2	17.2	408.2	0.0	337.0	129.4	0.0	0.0	0.0	0.0
Soil50/ Sand100	Ratio	4.35	3.37	NA	47.70	3.14	NA	0.73	6.90	21.64	1.39	NA	1.33	8.51	NA	NA	NA	NA

*Data within the columns with different letters are significantly different (Tukey tests; P < 0.05)*.

*Wild, wild emmer; Durum, durum wheat; Hexadec, hexadecanoic; Octadec, octadecanoic; 9-Octadec, 9-octadecenoic; Octacosan, octacosanol*.

NANOVA analysis was carried out separately for these two substrates (i.e., Soil50, Sand100) for each metabolite, to define the component of variance for evaluation of the heritability, and to estimate the mean divergence between the domestication groups for root exudates, measured as Q_ST_ (Table 3). With Soil50, the heritability for individual metabolites ranged from 2.67% for ribose to 78.1% for “mannose and glucose,” while with Sand100, it ranged from 19.6% for glycerol to 72.0% for oxalic acid.

**Table 3:** Heritability and Q_ST_ estimates for the mean metabolite levels for the Soil50 and Sand100 conditions.

**Metabolite**	**Growth substrate**			
	**Soil50**		**Sand100**	
	**Heritability (h^2^)**	**Q_ST_**	**Heritability (h^2^)**	**Q_ST_**
Fructose	58.73	0.79	56.99	0.19
Galactose	48.03	0.62	59.51	0.40
Isomaltose	42.29	0.03	–	–
Maltose + turanose	32.36	0.00	58.96	0.62
Mannose + glucose	78.10	0.69	62.94	0.23
Raffinose	26.48	0.00	–	–
Ribose	2.67	0.00	42.43	0.58
Sucrose	64.33	0.00	52.45	0.43
Glycerol	52.92	0.00	19.60	0.90
Mannitol	44.72	0.36	50.44	0.42
*myo*-Inositol	62.92	0.82	–	–
Sorbitol	41.69	0.26	63.91	0.52
Oxalic acid	36.95	0.00	72.02	0.00
Hexadecanoic acid	30.52	0.21	–	–
Octadecanoic acid	44.12	0.00	–	–
9-Octadecenoic acid	70.60	0.00	–	–
1-Octacosanol	77.57	0.03	–	–

The levels of genetic differentiation between domestication groups (i.e., the proportion of genetic variance between populations; Q_ST_) were calculated for each metabolite, to determine the differences among the substrates. For Soil50, these ranged from 0.03 for isomaltose and 1-octacosanol to 0.82 for *myo*-inositol, and for Sand100, from 0.19 for fructose to 0.90 for glycerol.

### Multivariate analysis

**Figure 3:**
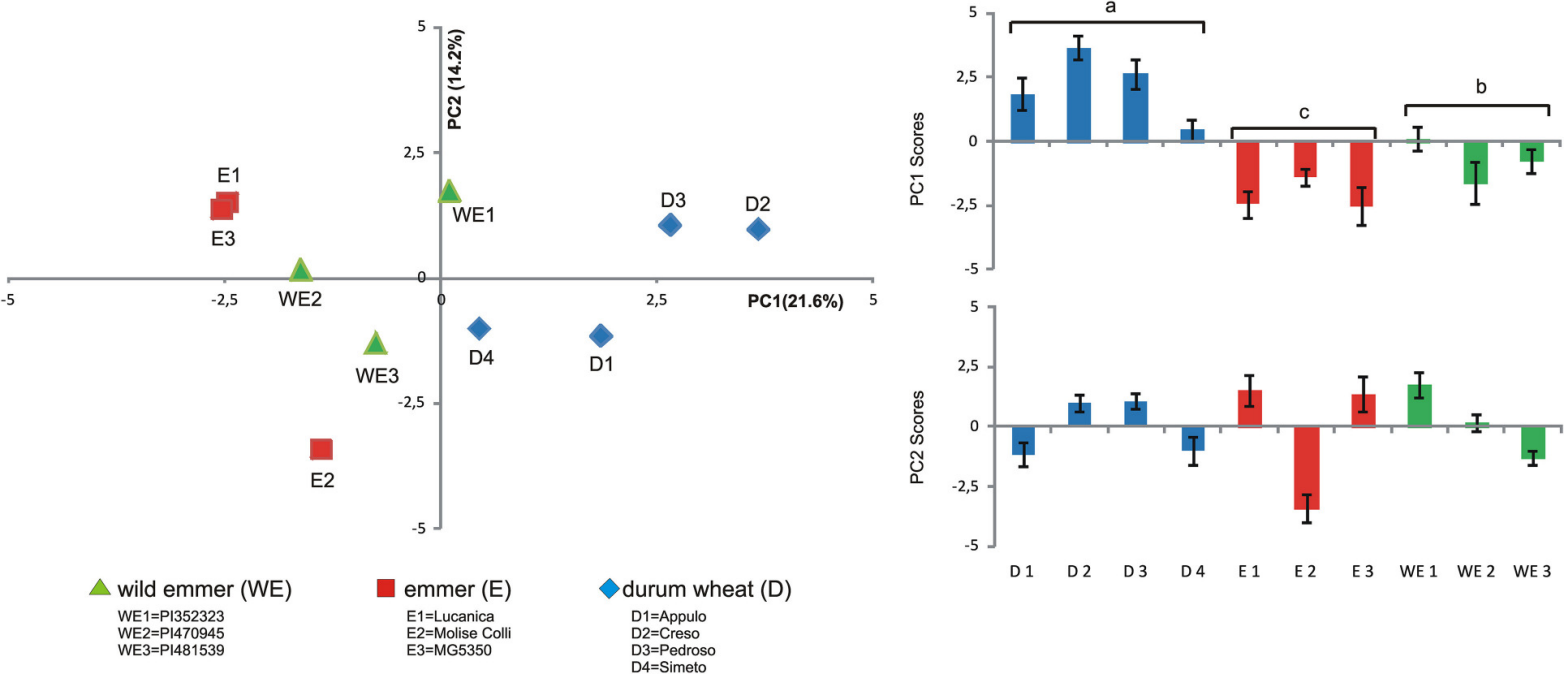
Principal component analysis of the metabolites of the root exudates associated with the different tetraploid wheats **(Left)**. Scores plot of the first and second axes (PC1, PC2, respectively) from the principal component analysis for the root exudate compositions of the different tetraploid wheats **(Right)**. WE, wild emmer; E, emmer; D, durum wheat.

Principal component analysis was also carried out for the root exudate metabolites using the whole replicated dataset of 10 genotypes, to determine whether overall the three domestication groups showed different patterns of metabolites. The PCA was performed using all the metabolites detected (Table 3), 17 out of 17 found for Soil50 and those found for Sand100 (10 out of 17), obtaining overall 27 independent variables for this analysis (Figure 3).

Considering the first six PCs (that explained at least 5% of the variance) together these explained 70.86% of the total variance estimated (Table S2). In particular, in Soil50, PC1 explained 21.57% of the total variability and was negatively correlated to *myo*-inositol and sugars (i.e., fructose, galactose, mannose, glucose), while in Sand100, PC1 was positively correlated to maltose, turanose and polyalcohols (i.e., mannitol, sorbitol, glycerol). In Soil50, the second component, PC2, explained 14.22% of the total variability, and was positively correlated to sucrose, raffinose, maltose, and turanose. Considering the loadings of these first two principal components, we highlight that the polar metabolites, and in particular the sugars and polyalcohols, are the main sources of diversity among these domestication groups (Table 3).

Considering Figure 3 (left), for the first two principal components, the genotypes of durum wheat were clearly well separated from the accessions of the two progenitor domestication groups (i.e., wild emmer, emmer), especially in the PC1 direction. This trend appeared clear, with durum wheat clustered differently from wild emmer and emmer due to the variability associated to the chemical diversity of the root exudates represented by PC1 (Figure 3, right). Similar behaviors were observed also when PCA was carried out considering the metabolite exudates for Soil50 and Sand100 separately. Figure S1 shows the score plots of the PCA of the metabolites of the root exudates associated with the different tetraploid wheat subspecies for soil50 (A, total variability explained by the first two components, 45.6%) and for sand100 (B, total variability explained by the first two components, 62.2%).

## Discussion

A preliminary observation that arises from the present study is that the soil type might dramatically affect both the plants (particularly in terms of root growth) and the composition of the root exudate in the rhizosphere. Indeed, root growth was highly stimulated here in the Sand100 substrate, which was similar to the effects of low nitrogen conditions on durum wheat (Gioia et al., 2015) and of drought in higher plants (Liu et al., 2017). This suggests that root growth in tetraploid wheat is under the control of a complex system that can promote growth under suboptimal conditions (e.g., of fertility, water). This behavior is often associated with sandy soils, with the main function being to increase the root:shoot ratio, and consequently the aptitude to absorb more water and nutrients from the deeper soil layers (Kuster et al., 2013; Taeger et al., 2015; Liu et al., 2017). Interestingly, these trends were much more pronounced here in the wild and domesticated emmer, compared to the modern durum wheat. The ability for the plants to respond differently to the availability of resources through promoting root growth under suboptimal conditions would be a very important adaptive trait in the wild, where germination can occur under much more diverse conditions compared to cultivated crops, where the farmers take care of the sowing (Fuller, 2007). The present study indicates that root morphology might be highly influenced by the effects of the environment, and that different soil conditions might promote major Genotype × Environment interactions. Thus, the soil conditions and their interactions with different genotypes need to be carefully considered prior to generalizing the phenotyping results of root traits obtained using high-throughput phenotyping systems as that described by Gioia et al. (2017).

For root exudation, Neumann et al. (2014) reported that in lettuce the soil type used for cultivation strongly influences the composition of the exudate. According to their findings, a similar situation was observed in the present investigation with wheat. In addition, considering the specific metabolites that were identified in the root exudates, it needs to be taken into account that the comparison here was between two different soil substrates that were also different in terms of their sterility. Indeed, while the Sand100 substrate was fully sterilized (including the seeds), the Soil50 substrate was obtained through the combination of sterilized sand and non-sterilized soil from fields. Thus, for Sand100, all of the metabolites observed were only direct plant products, while for Soil50, in addition to the direct effects of the plants, the potential effects of the interactions between the plants and the microbes in the rhizosphere need to be considered. Among other aspects, this might explain the observed pattern alone, thus showing strong differences between the soil types, and due to the interactions between genotype and soil type. Our results are in agreement with Kuijken et al. (2014) that indicate that sterile growth substrate is useful to test simple soil–plant interaction systems, and essential to investigate the quantity and composition of root exudates. Sterile growth substrate allows for a stable, reliable and easy phenotyping method, and can be used to investigate genetic and environmental effects on exudation. This is linked to the fact that sterilization of soil can result in major modifications to its structure, while in send sterilization is not expected to have similar effects.

The main result of the present study is that the composition of the rhizosphere metabolites is associated with differences among the genotypes of these wheat domestication groups, as can be seen by the high heritability of some of the metabolites. Indeed, this study is the first that has estimated the heritability of rhizosphere metabolites produced by plants. For many of the rhizosphere metabolites, significant differences were detected between these domestication groups within the soil types, with unexpectedly large heritability within soil type associated to a significant Genotype × Soil type interaction. This indicates the large levels of heritable variation within tetraploid wheat for determination of the composition of the rhizosphere metabolites.

However, a second observation of major importance from the present study is the significant genetic differentiation (Q_ST_) between these subspecies for many of the metabolites, which shows that the genotypic differences are largely associated with the key steps in the domestication of tetraploid wheat because of selection and drift. In particular, larger differences were associated with the development of durum wheat (secondary domestication). This correlation with the domestication processes might suggest an important adaptive nature of the changes seen in the root exudate compositions.

At the same time, by comparing heritability and Q_ST_ values, it is crucial to note the high level of heritable genotypic diversity between the genotypes within the subspecies. This suggests that root exudate production can be substantially modified by the selection imposed by the agroecosystem, or because of direct human selection. The influence of root exudates in shaping microbial communities associated with plant root systems has been receiving particular interest more recently (van Dam and Bouwmeester, 2016).

Considering the different chemical classes detected, it also needs to be noted that these metabolites might impact differently, and to different extents, upon the microbial communities associated with the rhizosphere. Even if it is generally true that active and passive activities lead to a carbon-rich environment in rhizosphere soil with respect to the bulk soil (Herrera Paredes and Lebeis, 2016), the patterns in the concentrations of the individual sugars (i.e., maltose, sucrose, raffinose) that are seen within the class of sugars are of particular importance for their possible influence on the rhizosphere microbiota. From this point of view, sucrose represents a good model for the potential wide impact of these individual sugars, as sucrose metabolism has pivotal roles in microbial cells due to its direct and indirect influences on growth, regulation of gene expression, stress responses, and signaling pathways (Ruan, 2014).

The other class of particular interest is the organic acids. Shi et al. (2011) noted that organic acids have added carbon, show improved solubilisation of soil organic matter, and promote changes in soil pH. Shi et al. (2011) demonstrated that potentially due to these aspects, organic acids caused significant increases in the detectable richness of the soil bacterial community and larger shifts in the dominant taxa, also when compared with the effects of the sugars. Further considering this sugars/ organic acids dichotomy, it is intriguing to note that when focussing on the specific plant-related functions of bacteria, such as nitrogen fixation, only sugar-containing substrates can directly induce the desired activity, and the presence of organic acids has been shown to have additional selective effects on the active diazotroph population (Bürgmann et al., 2005). All these aspects shed new light on the patterns of the individual metabolites that are shown here to be associated with key evolutionary steps of tetraploid wheat.

Furthermore, considering these two chemical classes of sugars and organic acids, the present study provides the molecular basis for further studies that can determine the impact of such individual metabolites on the shaping of the microbiota associated with the roots of tetraploid wheat. Indeed, we have to consider that in their analysis, Shi et al. (2011) monitored only the impact of the model solutions that contained the selected sugars (i.e., glucose, sucrose, fructose) and organic acids (i.e., quinic, lactic, maleic acids) that have previously been shown to be associated with the root exudates of the plant species studied. With this concern, similarities and differences have been reported for the root exudate compositions of *Brachypodium* (Kawasaki et al., 2016), which have been proposed as a promising model to investigate the microbiome of wheat.

More generally, our results support the hypothesis that root exudates (as affected by the genotype and by the genotype-by-environment interaction as soil type) can maintain and support a highly specific diversity of microbes in the rhizosphere associated with any given plant species. Mahoney et al. (2017), analyzed the community structure and species variation of rhizosphere-associated bacteria of different winter wheat cultivars, and demonstrated that the wheat cultivars differentially altered the bacterial abundances in the rhizosphere. This is in accord to the occurrence of soil-dependent and genotype-dependent variations in the composition of rhizosphere microbial communities (Lareen et al., 2016), and suggests close evolutionary links between these phenomena (Badri and Vivanco, 2009; Micallef et al., 2009; Neumann et al., 2014). Within this perspective, our data on the molecular phenotypes (i.e., metabolites) suggest that selection during wheat domestication and modern breeding might have had a major role in changes in microbiome–plant interactions, in agreement with evidence on barley domestication reported by Bulgarelli et al. (2015), through a genomic approach.

The present study adds a new milestone in the evaluation of the impact of plant domestication on the chemical diversity of the rhizosphere, including possible direct and indirect effects of these variations on the metabolite contents (Pérez-Jaramillo et al., 2016). Indeed, these data provide the first example that demonstrates the impact of domestication and crop evolution on rhizosphere composition, and in particular on the root exudate composition associated with wild emmer, domesticated emmer and modern durum wheat. Previous studies that suggested the role of domestication in barley were based on genomic comparisons of one wild barley genotype and one domesticated barley genotype, and they could not distinguish the effects of differences between populations compared with those between individuals within a population, and to associate the observed differences to differences between domestication groups (e.g., Poets et al., 2015). In addition to this effect, the present study shows high heritable variation also within subspecies, which suggests the potential for selecting target genotypes and populations that could improve the rhizosphere composition and, as a consequence that could promote environmental adaptation and agronomic performances. Moreover, this study constitutes the basis for further studies to investigate the effects of genotypic differences of root exudates in the microbiome community, and to explore the greater diversity compared to the small panel of genotypes used in the present study.

## Author contributions

RP, AI, MF, and RB conceived and designed the study; AI, MF, RB, and FN performed the experiments; RP, AI, MF, and RB analyzed the data; RP and MF drafted the manuscript; AI and RB contributed to the drafting and writing of the manuscript. All authors critically read and approved the manuscript.

### Conflict of interest statement

The authors declare that the research was conducted in the absence of any commercial or financial relationships that could be construed as a potential conflict of interest.

## Footnotes

^1^NIST Chemistry WebBook Available online at: http://webbook.nist.gov/chemistry/ (Accessed August 7, 2017).

## Supplementary material

Figure S1 Principal component analysis of the metabolites of the root exudates associated with the different tetraploid wheats in soil50 **(A)** and sand100 **(B)**. WE, wild emmer; E, emmer; D, durum wheat.

Table S1 Concentrations of individual metabolites detected in the substrate of the controls (i.e., pots with substrate but no plants) according to the Soil50 and Sand100 conditions.

Table S2 PCA for the metabolites associated with the 10 genotypes of the three cereal species grown in Soil50 and Sand100 and harvested at the third leaf developmental stage, showing the eigenvalues and the proportions of associated variation.
