# Supplementary material for: Evolution of the Crop Rhizosphere: Impact of Domestication on Root Exudates in Tetraploid Wheat (Triticum turgidum L.)
Source: Front Plant Sci. 2017 Dec 13;8:2124. doi: 10.3389/fpls.2017.02124 (PMC5733359; doi:10.3389/fpls.2017.02124)
Supplement: Table S1 — Concentrations of individual metabolites detected in the substrate of the controls (i.e., pots with substrate but no plants) according to the Soil50 and Sand100 conditions. [file TableS1.DOCX]

**Evolution of the crop rhizosphere: domestication of root exudates in tetraploid wheat (*Triticum turgidum* L.)**

Anna Iannucci^1,a^, Mariagiovanna Fragasso^1,a^, Romina Beleggia^1^, Franca Nigro^1^, Roberto Papa^1,2^*

**Table S1.** Concentrations of individual metabolites detected in the substrate of the controls (i.e., pots with substrate but no plants) according to the Soil50 and Sand100 conditions

| Control | Mannitol | Sorbitol | Myo-inositol | Sucrose | Maltose + turanose | Oxalic acid |
| --- | --- | --- | --- | --- | --- | --- |
| SOIL 50% | 47,5 | 38,75 | 7,5 | 1227.5 | 127973.8 | 166.3 |
| SAND 100% | - | - | - | 132.5 | 117.5 | 93.5 |
