# Supplementary material for: Evolution of the Crop Rhizosphere: Impact of Domestication on Root Exudates in Tetraploid Wheat (Triticum turgidum L.)
Source: Front Plant Sci. 2017 Dec 13;8:2124. doi: 10.3389/fpls.2017.02124 (PMC5733359; doi:10.3389/fpls.2017.02124)
Supplement: Table S2 — PCA for the metabolites associated with the 10 genotypes of the three cereal species grown in Soil50 and Sand100 and harvested at the third leaf developmental stage, showing the eigenvalues and the proportions of associated variation. [file TableS2.DOCX]

**Evolution of the crop rhizosphere: domestication of root exudates in tetraploid wheat (*Triticum turgidum* L.)**

Anna Iannucci^1,a^, Mariagiovanna Fragasso^1,a^, Romina Beleggia^1^, Franca Nigro^1^, Roberto Papa^1,2^*

**Table S2**. PCA for the metabolites associated with the 10 genotypes of the three cereal species grown in Soil50 and Sand100 and harvested at the third leaf developmental stage, showing the *eigenvalues* and the proportions of associated variation.

| **PC** | **Eigenvalue** | **PC**  **variation** | **Cumulative**  **variation** |
| --- | --- | --- | --- |
|  |  | **(%)** | **(%)** |
| 1 | 5.8248 | 21.573 | 21.573 |
| 2 | 3.8397 | 14.221 | 35.794 |
| 3 | 3.4187 | 12.662 | 48.456 |
| 4 | 2.1899 | 8.111 | 56.567 |
| 5 | 1.976 | 7.318 | 63.885 |
| 6 | 1.8834 | 6.975 | 70.861 |
| 7 | 1.1038 | 4.088 | 74.949 |
| 8 | 0.8976 | 3.325 | 78.273 |
| 9 | 0.7846 | 2.906 | 81.179 |
| 10 | 0.6668 | 2.470 | 83.649 |
| 11 | 0.6450 | 2.389 | 86.038 |
| 12 | 0.6132 | 2.271 | 88.309 |
| 13 | 0.4488 | 1.662 | 89.971 |
| 14 | 0.4377 | 1.621 | 91.592 |
| 15 | 0.3751 | 1.389 | 92.981 |
| 16 | 0.3298 | 1.221 | 94.203 |
| 17 | 0.2961 | 1.097 | 95.300 |
| 18 | 0.2681 | 0.993 | 96.293 |
| 19 | 0.2486 | 0.921 | 97.213 |
| 20 | 0.1616 | 0.599 | 97.812 |
| 21 | 0.1430 | 0.530 | 98.342 |
| 22 | 0.1293 | 0.479 | 98.820 |
| 23 | 0.1006 | 0.373 | 99.193 |
| 24 | 0.0815 | 0.302 | 99.495 |
| 25 | 0.0571 | 0.211 | 99.706 |
| 26 | 0.0447 | 0.165 | 99.872 |
| 27 | 0.0346 | 0.128 | 100 |
